# A Host-Vector System for the Expression of a Thermostable Bacterial Lipase in a Locally Isolated *Meyerozyma guilliermondii* SMB

**DOI:** 10.3390/microorganisms8111738

**Published:** 2020-11-06

**Authors:** Abu Bakar Salleh, Siti Marha Baharuddin, Raja Noor Zaliha Raja Abd Rahman, Thean Chor Leow, Mahiran Basri, Siti Nurbaya Oslan

**Affiliations:** 1 Enzyme and Microbial Technology Research Center, Universiti Putra Malaysia, 43400 UPM Serdang, Selangor, Malaysia; abubakar@upm.edu.my (A.B.S.); haakecik@gmail.com (S.M.B.); rnzaliha@upm.edu.my (R.N.Z.R.A.R.); adamleow@upm.edu.my (T.C.L.); mahiran@upm.edu.my (M.B.); 2 Institute of Bioscience, Universiti Putra Malaysia, 43400 UPM Serdang, Selangor, Malaysia; 3 Department of Microbiology, Faculty of Biotechnology and Biomolecular Sciences, Universiti Putra Malaysia, 43400 UPM Serdang, Selangor, Malaysia; 4 Department of Cell and Molecular Biology, Faculty of Biotechnology and Biomolecular Sciences, Universiti Putra Malaysia, 43400 UPM Serdang, Selangor, Malaysia; 5 Department of Chemistry, Faculty of Science, Universiti Putra Malaysia, 43400 UPM Serdang, Selangor, Malaysia; 6 Department of Biochemistry, Faculty of Biotechnology and Biomolecular Sciences, Universiti Putra Malaysia, 43400 UPM Serdang, Selangor, Malaysia

**Keywords:** *Pichia* sp., *Meyerozyma guilliermondii*, thermostable lipase, formaldehyde dehydrogenase promoter, yeast, alternative host

## Abstract

Screening for a new yeast as an alternative host is expected to solve the limitations in the present yeast expression system. A yeast sample which was isolated from the traditional food starter ‘ragi’ from Malaysia was identified to contain *Meyerozyma guilliermondii* strain SMB. This yeast-like fungus strain SMB was characterized to assess its suitability as an expression host. Lipase activity was absent in this host (when assayed at 30 °C and 70 °C) and Hygromycin B (50 μg/mL) was found to be its best selection marker. Then, the *hyg* gene (Hygromycin B) was used to replace the *sh ble* gene (Zeocin) expression cassette in a *Komagataella phaffii* expression vector (designated as pFLDhα). A gene encoding the mature thermostable lipase from *Bacillus* sp. L2 was cloned into pFLDhα, followed by transformation into strain SMB. The optimal expression of L2 lipase was achieved using YPTM (Yeast Extract-Peptone-Tryptic-Methanol) medium after 48 h with 0.5% (*v/v*) methanol induction, which was 3 times faster than another *K. phaffii* expression system. In conclusion, a new host-vector system was established as a platform to express L2 lipase under the regulation of P_FLD1_. It could also be promising to express other recombinant proteins without inducers.

## 1. Introduction

*Komagataella phaffii,* which was previously known as *Pichia pastoris,* is the most common host for recombinant protein expression in industries. Most foreign genes are expressed under the control of the *K*. *phaffii* alcohol oxidase 1 promoter (P_AOX1_), where methanol is a vital inducer. Some recombinant proteins require a longer time for optimal production and high methanol induction when expressed under the regulation of P_AOX1_ [1]. The negative effects of excessive methanol usage in this system have not been discussed much, since the recombinant proteins will normally be purified before use. Direct consumption of crude proteins is unsafe for human and feed industries. Thus, it is important to have a new yeast expression system from food sources as an alternative host with minimal enzymes production time to reduce the production cost and minimize the methanol toxicity effects.

Lipases are remarkable biocatalysts for high-value application in various industries [2]. Production of lipase from microorganisms has gained interest since this enzyme is used in numerous commercial applications. A lipase from thermophilic bacterium *Bacillus* sp. strain L2 (GenBank Accession No. AY855077) was isolated, purified, and characterized by Shariff et al. [3]. From research, the highest lipase activity was found to be only 0.89 U/mL after culturing in a medium at pH 7 containing casamino acid, trehalose, Ca^2+^ and Tween 60 at 70 °C and 150 rpm with 1% inoculum size. However, Sabri [4] showed that the highest L2 lipase expression (91 U/mL and 125 U/mL for pPαS3 and pPαG2, respectively) in *K. phaffii* was at 144 h with 6 times methanol induction [0.5% (*v/v*)] every 24 h.

Several tools encompassing the collection of host strains, vectors with recessive and dominant markers, and several transformation protocols have been developed for molecular genetic studies in yeast-like fungi *Meyerozyma guilliermondii* [5]. A recent study proved that the *M. guilliermondii* strain SO can express the recombinant T1 lipase under the regulation of P_AOX1_. The host managed to reduce the methanol usage at 5-fold as compared to *K. phaffii* [6] and the time needed was only 30 h with 1.5% (*v/v*) methanol induction [7]. In addition, Abu et al. [8] reported that the yeast is also capable of expressing the recombinant protein without methanol. The exploitation of strain SO recently proved that it could express a bacterial enzyme at a significant level under formaldehyde dehydrogenase 1 promoter (P_FLD1_) regulation without any inducers [9]. This finding proved that *M. guilliermondii* may have been auto-induced during cell cultivation by its by-product, which has not yet been determined. Considering the fact that *M. guilliermondii* has significant value as a host from recombinant protein expression, this research highlights the isolation and identification of a locally isolated yeast (strain SMB) to be used as an alternative host for bacterial lipase expression using a modified vector system. This study involves the isolation of the yeast sample from a traditional safe food consumed by the Southeast Asia population.

## 2. Materials and Methods

### 2.1. Strains and Plasmid

Local yeast isolate (strain SMB) collected from a traditional fermented food starter (ragi) in Malaysia was cultured in YPD medium [Yeast Peptone Dextrose: 1% (*w/v*) yeast extract (Becton Dickinson, Franklin Lakes, NJ, USA), 2% (*w/v*) peptone (Becton Dickinson, Franklin Lakes, NJ, USA), 2% (*w/v*) agar (Becton Dickinson, Franklin Lakes, NJ, USA), and 2% (*w/v*) dextrose (Chemiz, MY)]. The recombinant strain SMB and *Komagataella phaffii* strain GS115 (pPαG2) containing recombinant L2 lipase were grown in YPD supplemented with Hygromycin B (InvivoGen, San Diego, CA, USA) (50 μg/mL) and Zeocin (InvivoGen, San Diego, CA, USA) (100 μg/mL), respectively. *Escherichia coli* JM109 harbouring pFLDα and *E. coli* containing DH5α pSELECT-hygro-mcs plasmids were grown in Luria-Bertani medium supplemented with 50 μg/mL ampicillin (Sigma Aldrich, St. Louis, MO, USA). The vectors were purchased from Invitrogen, USA and InvivoGen, USA, respectively.

### 2.2. Identification of Locally Isolated Yeast Strain SMB

Yeast sample was collected from the traditional fermented food starter, ‘*ragi*’. The sample was spread on a YPD plate supplemented with 50 mg/mL ampicillin after serial dilutions were done. Then, the plates were incubated at 30 °C for 2 days until colonies formed. Then, the yeast-like colonies were selected and sub-cultured on the YPD plate.

Next, a yeast-like single colony was inoculated into 10 mL YPD broth, followed by extracting the total genomic DNA using DNeasy Blood and Tissue Kit (Qiagen, Hilden, Germany). The amplification of the ITS region was carried out in 20 μL of reaction mixture containing i-Taq PCR premix, 16 μL of distilled water, 1 μL each of 10μM forward (fITS1) and 10 μM reverse (rITS4) primers (refer to Table 1); and 2 μL (10 ng) of genomic DNA template. The amplification was performed with a total of 36 polymerase chain reaction (PCR) cycles using a thermal cycler (Bio-Rad, Hercules, CA, USA). The reaction was performed at the following parameters: pre-denaturation at 94 °C for 10 min, 36 repeated cycles for denaturation at 94 °C (30 s), annealing at 60 °C (30 s), and extension at 72 °C (1 min). The final extension was run at 72 °C for 7 min. The similar PCR mixture was used to identify strain SMB using 18S rRNA (f18S, r18S) and 25S rRNA (f25S, r25S) primers. Table 1 shows the list of primers used in this study. The amplicons were sequenced (First BASE Laboratories Sdn. Bhd.), the results were analyzed using the Biology Workbench 2.0 (http://workbench.sdsc.edu/), and the species identified using the Basic Local Alignment Search Tool (BLAST) at www.ncbi.nlm.nih.gov/BLAST/.

### 2.3. Screening of Native Lipase in Strain SMB

Strain SMB underwent lipase screening procedures prior to utilizing it as a host for bacterial L2 lipase expression. For the quantitative assay, lipase activity was measured colourimetrically by the method described by Kwon and Rhee [14]. The substrate emulsion was prepared by mixing an equal volume [1:1 (*v/v*)] of olive oil (Bertoly, IT) and 50 mM phosphate buffer (pH 7.0) with a homogenizer until it was emulsified. The reaction mixture (20 μL of 0.02 mM CaCl_2_, 2.5 mL substrate emulsion, 0.5 mL enzyme, and 0.5 mL phosphate buffer) was shaken for 30 min at 30 °C and 70 °C, separately. Then, the reaction was stopped by the addition of 1.0 mL of 6 M HCL and 5.0 mL isooctane (Merck, DE) and vigorously vortexed for 1 min. The upper layer (3.0 mL) containing the fatty acids was transferred to another test tube consisting 1.0 mL of 5% (*w/v*) copper pyridine for free fatty acids colour detection. Then, the mixture was vortexed and left to settle for 30 min. Finally, 1.0 mL of the upper layer isooctane was taken for OD_715nm_ reading using a UV-visible spectrophotometer (Ultra Spec 2100 Pro, Amersham Bioscience). Unit (U) definition for lipase activity was defined as the rate of fatty acid formation (μmole) per min (standard assay condition).

### 2.4. Determination of Antibiotic Selection Markers

A variety of antibiotics was added to the YPD agar to assess the antibiotic resistance for strain SMB. A single colony was inoculated into 10 mL YPD broth (incubated at 30 °C with 250 rpm) and 100 µL of the overnight yeast culture was spread on several YPD plates supplemented with different types of antibiotics at respective concentrations for 3 days. The antibiotics and the recommended concentrations used were as follows: Blasticidine-S (InvivoGen, San Diego, CA, USA) (50 μg/mL), Zeocin (500 μg/mL), Hygromycin B (50 μg/mL), Puromycin (InvivoGen, San Diego, CA, USA) (25 μg/mL), Geneticin (InvivoGen, San Diego, CA, USA) (400 μg/mL), and Phleomycin (InvivoGen, San Diego, CA, USA) (25 μg/mL).

### 2.5. Amplification of Methanol-Inducible Promoters

The presence of methanol-inducible promoters in the local isolate was screened using PCR. Two methanol-inducible promoters tested were alcohol oxidase 1 (P_AOX1_), and formaldehyde dehydrogenase 1 (P_FLD1_). Amplification of the genes was performed using the specific promoter primers, as listed in Table 1. The amplification was carried out in 25 μL of reaction mixture containing 12.5 μL 2× PCR premix (Bioline, UK), 9.5 μL of distilled water, 1 μL each of (10 μM) forward and (10 μM) reverse primers, and 1 μL (10 ng) of DNA template. The amplification was performed using a standard PCR condition with different annealing temperatures for P_AOX1_ (51 °C) and P_FLD1_ (50 °C).

### 2.6. Construction of Recombinant pFLDhα/L2 Lipase

pFLDα vector with *sh ble* gene deletion was amplified using the PCR. The primers used were forward (CYC1 *Hind*III F) and reverse (pTEF1 *Pst*I R). The amplified pFLDα without the *sh ble* gene was designated as pFLDα-Z. The pFLDα-Z was amplified using PCR in a 50 μL reaction mixture containing 10 μL of 5× Hi-Fi buffer, 0.5 μL of 100 mM dNTP, 1 μL (20 pmol/μL) of each forward and reverse primers, 3 U of VELOCITY DNA polymerase, 2 μL of plasmid DNA (100 ng/μL), 2 μL of DMSO, and topped up with distilled water. The gene was amplified in 30 PCR cycles with the following parameters: pre-denaturation at 96 °C for 2 min, 30 repeated cycles for denaturation at 96 °C (30 s), annealing at 64 °C (30 s), and extension at 72 °C (1 min). The final extension was run at 72 °C for 7 min. Then, the Hygromycin B (*hyg*) gene (CMV-em7-*hyg*) was amplified from pSELECT-hygro-mcs vector in 50 μL reaction mixture using forward (*hyg*-cmvF) and reverse (*hyg*-cmvR) primers, respectively. The same PCR mixture and condition were used to amplify the *hyg* gene, except that the annealing temperature was set at 66 °C and the final extension time was 24.5 min.

Next, both pFLDα-Z and CMV-em7-*hyg* were digested with *Hind*III and *Pst*I followed by overnight ligation using DNA T4 ligase at 16 °C. Then, the ligation mixture was transformed into *E. coli* JM109 according to the method suggested by Sambrook et al. [15]. The modified pFLDα-Z with the CMV-em7-*hyg* was designated as pFLDhα (5.9 kb). The gene encoding mature L2 lipase was amplified using PCR from the recombinant K. *phaffii* strain GS115 genome harbouring pPICZαA/L2 lipase (pPαG2) using forward (f*Sfi*IL2) and reverse (r*Kpn*IL2) primers [4]. The following conditions were used to amplify the *L2 lipase* gene from the recombinant: pre-denaturation at 94 °C for 4 min, 30 repeated cycles for denaturation at 94 °C (1 min), annealing at 65 °C (2 min), and extension at 72 °C (1 min). The final extension was run at 72 °C for 7 min.

Next, the pFLDhα and *L2 lipase* gene were digested with *Sfi*I and *Kpn*I prior to ligation at 16 °C with T4 ligase overnight. Then, the ligation mixture was transformed into *E. coli* DH5α and spread on Luria Bertani (Becton Dickinson, Franklin Lakes, NJ, USA) ampicillin (50 μg/mL) plate. The recombinant plasmid was designated as pFLDhα/L2lipase. Then, the pFLDhα/L2 was linearized by *Nde*I, followed by transformation into strain SMB onto the YPD Hygromycin (50 μg/mL) plate using the LiAc treatment method (EasySelect^TM^*Pichia* Expression Kit Manual Invitrogen, Carlsbad, CA, USA). The positive transformants were screened via colony PCR using forward (α-factor) and reverse (3′ AOX1) primers with annealing temperature of 60 °C.

### 2.7. Expression of L2 Lipase in Recombinant M. guilliermondii SMB

A single colony of the recombinant strain SMB carrying the pFLDhα/L2 was inoculated in 10 mL YPD broth at 30 °C and 250 rpm overnight. Then, 100 μL of the culture were inoculated into 100 mL of MGAs [0.17% (*w/v*) YNB (Becton Dickinson, Franklin Lakes, NJ, USA), 0.5% (*w/v*) ammonium sulphate (Merck, GE), 1% (*v/v*) glycerol (Merck, DE), 4 × 10^−5^% (*w/v*) biotin (Sigma Aldrich, St. Louis, MO, USA)], and cultivated under the same condition until OD_600nm_ = 4. Then, the cells were harvested at 3000× *g* and room temperature (RT) for 5 min. The cell pellet was resuspended in 20 mL MMAs [same composition as MGAs with the exception that glycerol was replaced with 0.5% (*v/v*) methanol (Merck, DE)] and cultivation was continued for 5 days. Methanol [0.5% (*v/v*)] was added every 24 h for induction. The strain SMB containing the empty vector (pFLDhα) was used as the control. Aliquots (15 mL) of the cultures were harvested at 8000× *g* and RT for 3 min every 24 h of post-induction. Finally, the supernatant was assayed colourimetrically using the previously described method at 70 °C. Recombinant strain SMB/pFLDhα/L2lipase (pFαS3) with the highest lipase expression was selected for further optimization.

### 2.8. Optimization of L2 Lipase Production

The effect of different medium formulations was studied using three different media. Recombinant strain (pFαS3) culture from the YPD medium was inoculated into 100 mL MGAs, BMGY [1% (*w/v*) yeast extract, 2% (*w/v*) peptone, 100 mM potassium phosphate, 1.34% YNB, 4 × 10^−5^% (*w/v*) biotin, 1% (*v/v*) glycerol], and YPTG [1% (*w/v*) yeast extract, 2% (*w/v*) peptone, 4 × 10^−5^% (*w/v*) biotin, 1% (*v/v*) glycerol, 2% (*w/v*) tryptone soy broth (Becton Dickinson, Franklin Lakes, NJ, USA)] media. Then, the cells were cultivated until the culture reached an OD_600nm_ = 4. Next, the cells were harvested at 3000× *g* and RT for 5 min followed by resuspension to a final volume of 100 mL in induction media MMAs, BMMY [same composition as BMGY with the exception the glycerol was replaced with 0.5% (*v/v*) methanol], and YPTM [same composition as YPTG with the exception that glycerol was replaced with 0.5% (*v/v*) methanol], respectively. Methanol [0.5% (*v/v*)] was added every 24 h and cultivation was stopped after 48 h. Cell growth was monitored and the supernatant from each flask was assayed for lipase activity for the optimization of each factors.

For the effect of different types of inducers, recombinant pFαS3 culture was grown in 100 mL MGAs. Then, the cells were harvested and resuspended in 20 mL of MMAs medium (for induction with methanol), MGMa medium [0.17% (*w/v*) YNB, 4 × 10^−5^% (*w/v*) biotin, 1% (*v/v*) glycerol, 0.25% (*w/v*) methylamine (Merck, DE)] (for induction with methylamine) and MMMa medium [same composition as MGMa with the exception that glycerol was replaced by 0.5% (*v/v*) methanol] (for induction with methanol plus methylamine), respectively. The respective inducers were added every 24 h for 48 h cultivation.

Next, the effect of different inducer concentrations was investigated. Recombinant pFαS3 culture was grown in YPTG, followed by YPTM media. During the induction phase, different methanol concentrations (0% 0.25%, 0.5%, 0.75%, 1%) were used to induce the cultures every 24 h. At 48 h, the cultivation was stopped, followed by lipase assay and growth measurement (at OD_600nm_). The effect of different growth temperatures on L2 lipase production was studied at 20 °C, 25 °C, 30 °C, and 35 °C. Recombinant pFαS3 culture was grown in 100 mL of YPTG, followed by YPTM supplemented with 0.5% (*v/v*) methanol every 24 h for 48 h in respective temperatures. Finally, for time-course study, the recombinant pFαS3 culture was grown in YPTM medium supplemented with 0.5% (*v/v*) methanol at 25 °C for 120 h. A 5 mL culture was harvested every 24 h for lipase assay and cell growth was monitored. Then, 12% SDS-PAGE [16] was run to monitor the size of His-tagged fusion of L2 lipase secreted into the medium. The samples were concentrated up to 10 times using spin column AMICON ULTRA-15 15ML (Merck, DE)—10 kDa cut-off. Then, both non-concentrated (2.8 µg) and concentrated (28.2 µg) crude enzymes were loaded in the gel.

## 3. Results and Discussion

### 3.1. Identification and Characterization of Strain SMB

**Figure 1:**
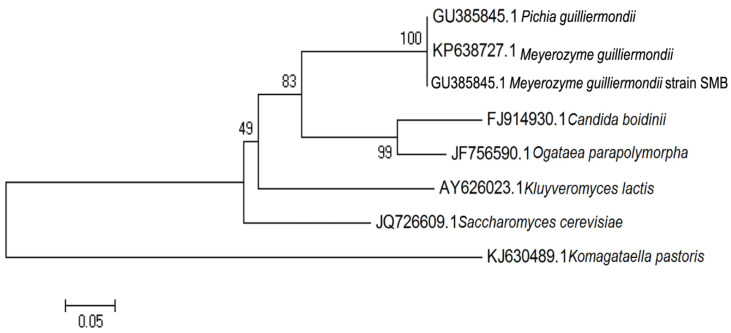
Evolutionary relationships of sample SMB using internal transcribed spacer (ITS) rDNA region. GU385845.1 *Pichia guilliermondii*, KP638727.1 *Meyerozyma guilliermondii*, GU385845.1 *Meyerozyma guilliermondii* strain SMB, FJ914930.1 *Candida boidinii*, JF756590.1 *Ogataea parapolymorpha*, AY626023.1 *Kluyveromyces lactis*, JQ726609.1 *Saccharomyces cerevisiae*, KJ630489.1 *Komagataella pastoris*.

Molecular systematics are very useful in identifying a yeast sample at the species level [17]. From the sequencing analysis, the full sequence of ITS rRNA local isolate strain SMB was obtained with length of 610 bp (Genbank accession number: GU385845.1). The BLAST results showed that the sequence was identical to *M. guilliermondii*. For years, the heterothallic and sporogenous yeast, *M. guilliermondii,* has been used in riboflavin production. The evolutionary relationships of strain SMB using rDNA ITS region are illustrated in Figure 1.

### 3.2. Screening of Strain SMB Native Lipase

The most important criterion was to ensure that the host did not possess any native lipase. The results showed that strain SMB did not exhibit any lipase activity in both intra and extracellular fractions at 30 °C and 70 °C. This finding proved that the strain could be used as a host without interfering with the expression of the desired thermostable bacterial lipase. In addition, although yeasts, mould, and bacteria produce comparable amounts of lipases, bacterial lipases are more widely used in industrial applications [18]. Strain SMB was chosen because it was found to be a species under *Pichia* genera with the expectation to have an ability to be the expression host just like the commonly used *K. phaffii*, *Ogataea polymorpha,* and *Ogataea methanolica* [19,20]. Further molecular identification was conducted to validate strain SMB. 18S rRNA and 25S rRNA sequencing results showed that this isolate belonged to *Debaryomyces hansenii* and *M. guilliermondii*, respectively. A study reported by Mota et al. [21] showed that these two species were closely related. Thus, in this study, strain SMB was assigned as *M. guilliermondii* strain SMB.

### 3.3. Determination of Antibiotic Selection Marker

In this study, *M. guilliermondii* strain SMB was tested on the YPD plate containing six types of antibiotics (Table 2). The result showed that hygromycin (50 μg/mL) was the best selection marker for strain SMB. A similar result was reported in *M. guilliermondii* strain SO where hygromycin (50 μg/mL) could be used to inhibit its growth [11]. Hygromycin is a commonly used antibiotic for inhibiting polypeptide synthesis, where it blocks the translocation steps in 80S ribosome in eukaryotic cells. However, strain SMB was able to survive in zeocin up to 500 μg/mL, where 100 μg/mL is normally used to select the positive transformant in *K*. *phaffii*.

### 3.4. Screening of Methanol-Inducible Promoters

**Figure 2:**
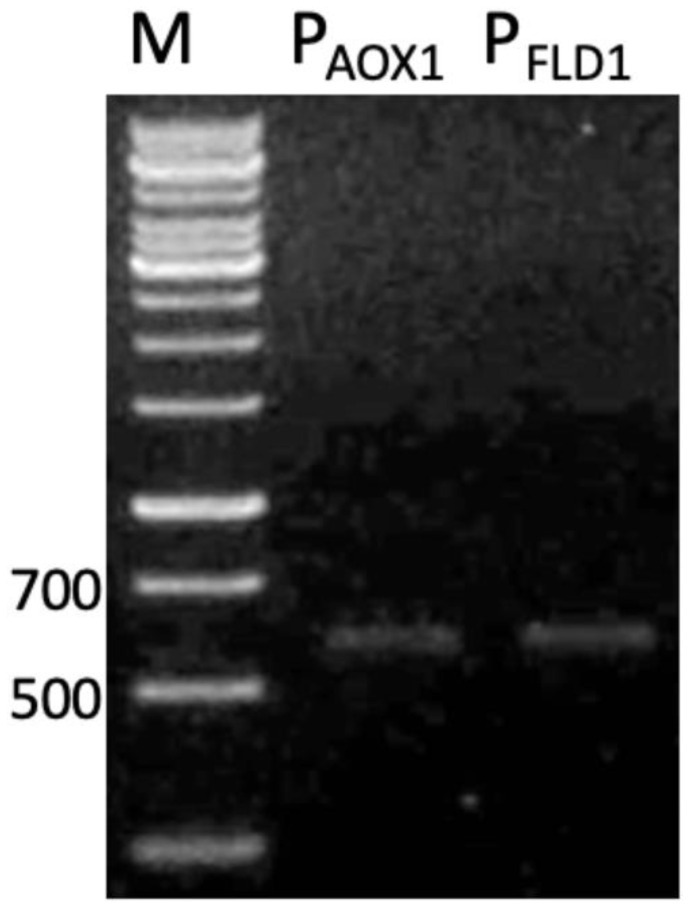
Gel electrophoresis of promoters amplification using different sets of primers. The PCR products were electrophoresed on 1% (*w/v*) agarose gel and stained with GelRed (Merck, DE). M: 1kb DNA ladder, P_AOX1_: alcohol oxidase 1 promoter; P_FLD1_: formaldehyde dehydrogenase 1 promoter.

The applications of methylotrophic hosts such as *Pichia* genera for foreign protein expression are linked to the use of strong methanol-inducible promoters derived from genes of the methanol utilization pathway, such as alcohol oxidase 1 (AOX1), and formaldehyde dehydrogenase 1 (FLD1). The availability of the promoters for these genes were investigated in strain SMB. The amplified P_FLD1_ and P_AOX1_ using the designed primers is shown in Figure 2, displaying the partial size of the amplified P_AOX1_ (550 bp) over its full sequence (941 bp). In this study, P_FLD1_ was chosen to be used as a promoter to regulate the recombinant L2 lipase expression in strain SMB. *FLD1* gene is the key enzyme required for the catabolism of methanol as a carbon source and methylamine as nitrogen sources in methylotrophic yeasts [22]. In addition, the *FLD1* gene is useful for regulating the expression of foreign genes under conditions where methanol is inappropriate. A sequence of P_FLD1_ from strain SMB showed 99% similarity to P_FLD1_ from *K. phaffii.*

### 3.5. Optimization of L2 Lipase Production in Strain SMB

**Figure 3:**
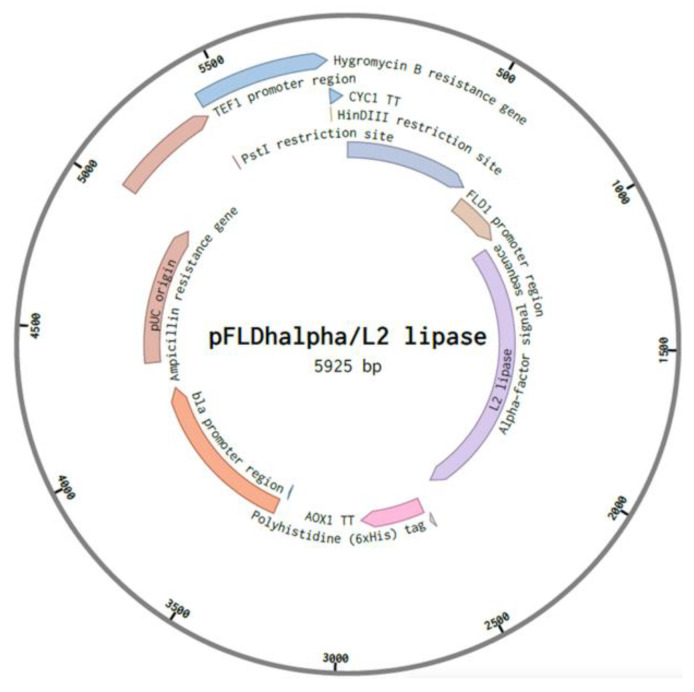
Plasmid construction for recombinant protein expression in strain SMB. The hygromycin expression cassette and *L2 lipase* gene were cloned into the plasmid, forming pFLDhα/L2 lipase.

**Figure 4:**
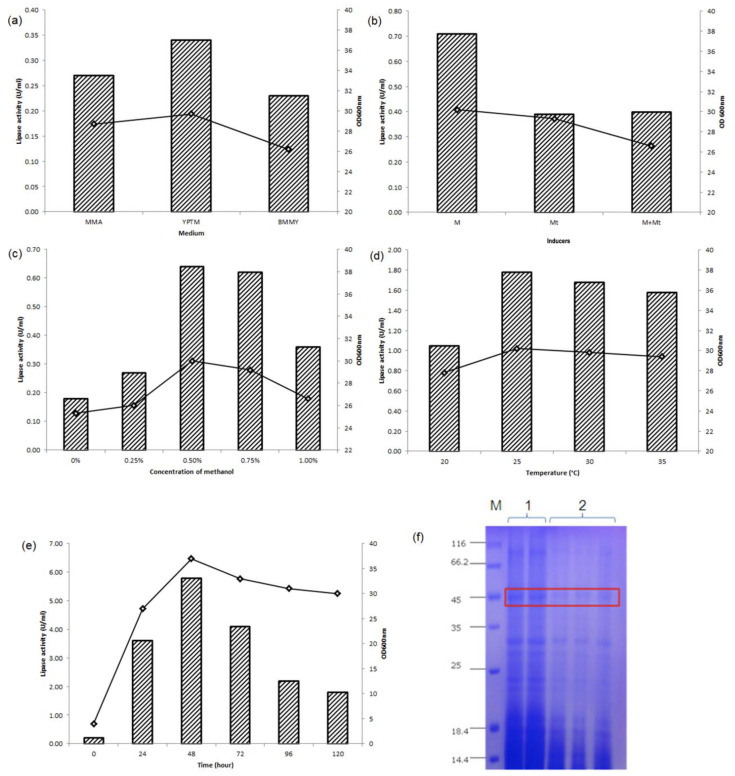
Optimization of L2 lipase expression: (**a**) Effect of various media on yeast growth and L2 lipase expression. Effect of media on L2 lipase expression at 48 h of cultivation of recombinant strain SMB when induced with 0.5% (*v/v*) of methanol. MMAs [Minimal Methanol with Ammonium sulphate], MGMa medium [Minimal Glycerol with Methylamine], MMMa medium [Minimal Methanol and Methylamine], BMMY [Buffered Minimal Methanol Yeast extract], and YPTM [Yeast extract, Peptone, Tryptic soy broth, biotin, Methanol]; (**b**) Effect of different types of inducer on L2 lipase expression in YPTM medium. Effect of inducers on lipase expression at 48 h of cultivation of recombinant strain SMB when induced with 0.5% (*v/v*) of methanol (M), 0.25% (*v/v*) methylamine (Mt) or both (M + Mt); (**c**) Effect of inducer concentration on L2 lipase expression in YPTM medium. Recombinant strain SMB was induced with different concentrations of methanol (0, 0.25, 0.50, 0.75, 1.0%) for 48 h; (**d**) Effect of temperature on L2 lipase expression in YPTM medium. Recombinant strain SMB was grown at different temperatures (20, 25, 30, 35 °C) and induced with 0.5% (*v/v*) methanol for 48 h; (**e**) Time course study of recombinant L2 lipase expression of recombinant strain SMB in YPTM medium at 25 °C for 120 h; (**f**) SDS-PAGE analysis of culture pFαS3. M: standard protein markers; Lane 1: concentrated crude enzyme (28.2 µg); Lane 2: non-concentrated crude enzyme (2.8 µg). Bars indicate enzyme activity (U/mL), while plots indicate OD_600nm_. (Data are presented as ±SD of triplicates.) Cell growth and lipase activity were monitored throughout the experiment.

Out of 20 positive clones, recombinant 3 (assigned as pFαS3) exhibited the highest activity when the *L2 lipase* gene was cloned into pFLDhα (Figure 3). Thus, it was selected for further optimization. Figure 4a shows the biomass and L2 lipase production in each medium at 48 h post-induction. The induction media affected both the biomass generation of recombinant strain SMB and the production of L2 lipase. Moderate lipase activities were observed when a minimal medium (MMA) was employed (0.27 U/mL). Furthermore, the additions of peptide-rich supplements such as yeast extract (BMMY) and tryptone soy broth and peptone (YPTM) to the expression media resulted in an efficient lipase secretion when methanol was used as a sole carbon source with 0.23 U/mL and 0.34 U/mL, respectively.

All three types of inductions were compared to assess the best inducer for *Pichia* transformants. From Figure 4b, 48 h cultivation induced by methanol gave the highest L2 lipase expression with 0.71 U/mL, followed by methanol plus methylamine (0.4 U/mL). Lipase production induced by methylamine gave the lowest production (0.39 U/mL). Our data suggested that methanol (carbon source) functioned as the best inducer compared to nitrogen. Figure 4c shows that 0.5% (*v/v*) methanol gave the highest expression (0.64 U/mL) with the optimum cell biomass. Increasing the methanol concentration resulted in increased expression level, up to the optimum methanol concentration; whereby too much supply showed an inhibition effect with a decrease in expression level. Significant lipase activity was also detected in the absence of methanol. A similar trend was reported by Abu et al. [8], where *M. guilliermondii* strain SO could also express the recombinant lipase without methanol.

Figure 4d shows that the recombinant strain SMB exhibited its optimal growth at 25 °C with 1.77 U/mL of lipase activity. It was observed that too low and too high growth temperature were not suitable for yeasts, which grow optimally at 30 °C. From the optimization study, it could be concluded that L2 lipase expression in strain SMB gave maximum expression at 25 °C, which was lower than the yeast optimum temperature (30 °C). The time course study of pFαS3 is shown in Figure 4e. Within 0 h to 48 h, the cell growth increased gradually coupled with lipase activity up to 5.7 U/mL but decreased by 120 h. These results showed that the L2 lipase expression in strain SMB was growth associated. The highest expression of L2 lipase was observed at the optimal growth of strain SMB (OD_600nm_ = ~37).

Referring to Figure 4f, the secreted His-tagged fusion L2 lipase showed a clear band at the expected size (~45 kDa). However, only 0.006 mg/mL of crude protein was secreted by the empty host, where it was insufficient to be detected via Coomassie staining. The new host-vector system has proven to produce L2 lipase optimally at 48 h, which was 3 times faster than that of *K. phaffii* (144 h) [23]. Further optimization strategies such as Response Surface Methodology (RSM) could be performed to improve the recombinant L2 lipase expression. A comparable result was also observed in *M. guilliermondii* strain SO, where the T1 lipase activity was optimally produced after 30 h of cultivation in YPTM with 1.5% (*v/v*) methanol [7]. Optimization without methanol induction could also be done to eliminate the use of methanol, where the secreted L2 lipase could be safely used in food or feed industries. Nonetheless, Nasir et al. [9] also reported on the use of P_FLD1_ in *M. guilliermondii* strain SO to express the recombinant protein without the presence of an inducer, where its production was subsequently optimized. Similar optimization strategies could also be performed for strain SMB in expressing L2 lipase.

## 4. Conclusions

In this study, *M. guilliermondii* strain SMB was identified as a new host isolated from a traditional food starter; it was developed as a new host-vector system for bacterial enzyme expression. The newly developed host-vector system demonstrated the ability to express bacterial L2 lipase extracellularly, three times faster than a commercial yeast system. It was also suggested that this system could be used as an alternative workhorse for other recombinant protein expression with and without methanol induction.

## Figures and Tables

**Table 1:** List of primers used in this study. T_A_ = Annealing temperature; AOX = alcohol oxidase; FLD = formaldehyde dehydrogenase.

Targeted Regions	Primers	Nucleotide Sequence (5′→3′)	T_A_ (°C)	Reference
Identification of isolates				
rRNA ITS	fITS1 rITS4	TCCGTAGGTGAACCTGCGG TCCTCCGCTTATTGATATGC	60.0	[10]
rRNA 18S	f18S r18S	AACCTGGGTTGATCCTGCCAGT TGATCCTTCTGCAGGTTCACCTAC	65.0	[11]
rRNA 25S	f25S r25S	TCATGAGACTACTGGCAGGATCAAC GGATCCGTTTAGACCGTCGTGAGA	64.7	[12]
Promoters				
AOX 1	AOX1f AOX1r	GACTGGTTCCAATTGACAGC GCAAATGGCATTCTGACATCC	51	[13]
FLD 1	FLD1f FLD1r	CGGGATCCGCATGCAGGAATCTCTGGA CGCAATTGTGTGAATATCAAGAATTG	50	Invitrogen, USA
Vector modification				
pFLDα-Z	*CYC*1 *Hind*III F pTEF1 *Pst*I R	ATACAAGCTTCACGTCCGACGCGGCCCGACGGGT TATACTGCAGCCGCCCTTAGATTAGATTGCTATGCTTTCT	66	This study
Hygromycin gene	*hyg*-cmvF *hyg*-cmvR	TTACCTGCAGGCGTTACATAACTTACGGTAAATGG AATCAAGCTTTCATTCCTTTGCCCTCGGACGAGT	64	This study
Cloning of L2 lipase				
Mature L2 lipase	f*Sfi*I L2 r*Kpn*I L2	AATTGGCCCAGCCGGCCAGCATCCCTA TTCTAGGTACCAGGGAGCAAGCTTGCCAA	65	This study
α-factor/3′AOX1	α-factor 3′ AOX1	TACTATTGCCAGCATTGCTGC GCAAATGGCATTCTGACATCC	60	Invitrogen, USA

**Table 2:** Determination of the drug selectable markers for strain SMB. (+++), (+), and (-) indicate high, low, and no growth, respectively.

Markers	Concentration (μg/mL)	Growth
Blasticidin	50	+++
Puromycin	25	+++
Geneticin	400	+++
Phleomycin	25	+++
Zeocin	500	+
Hygromycin	50	-
